# Unveiling the cellular and molecular mechanisms of diabetic retinopathy with human retinal organoids

**DOI:** 10.1038/s41419-025-08244-1

**Published:** 2025-12-19

**Authors:** Lada Polešovská, Simona Trmačová, Canan Celiker, Eva Hrubá, Francisco Molina Gambin, Václav Chochola, Veronika Matušková, Eleni Beli, Tomáš Bárta

**Affiliations:** 1 Department of Histology and Embryology, Faculty of Medicine, Masaryk University, Brno, Czech Republic; 2 Department of Ophthalmology, University Hospital Brno and Faculty of Medicine, Brno, Czech Republic; 3 Wellcome Wolfson Institute for Experimental Medicine, Queen’s University Belfast, Belfast, United Kingdom

**Keywords:** Experimental models of disease, Induced pluripotent stem cells, Mechanisms of disease

## Abstract

Diabetic retinopathy (DR) is a leading cause of vision impairment worldwide, driven by chronic hyperglycaemia and its complex metabolic consequences. While animal models have been widely used to study DR, they often fail to replicate the physiology of the human retina. Here, we employed human retinal organoids to investigate the effects of incremental hyperglycaemic stress—a modest increase from a standard high-glucose baseline (17.5 mM) to 25 mM D-glucose—across different stages of retinal differentiation. Early-stage organoids demonstrated resilience to high-glucose levels, maintaining normal morphology, viability, and gene expression. In contrast, late-stage organoids exhibited marked photoreceptor vulnerability, including downregulation of outer segment (OS)-specific genes, shortened OSs, increased oxidative stress, astrocyte activation, and significantly higher levels of apoptosis. Transcriptomic analysis revealed substantial changes in pathways related to vision, including the G protein-coupled receptor signalling pathway, response to light stimulus, and visual perception. While photoreceptors were particularly vulnerable, other retinal cell types, including bipolar cells, ganglion cells, and Müller glia, showed greater resilience. Additionally, glial activation, evidenced by increased expression of astrocyte markers, suggested an adaptive response to hyperglycaemia. To validate our findings, we compared our dataset with publicly available transcriptomic datasets from human retinas with DR, confirming key overlaps in pathways related to photoreceptor dysfunction, gliogenesis, and oxidative stress responses. While this non-vascularised model does not replicate the onset of DR from physiological glucose levels, it provides a human-specific platform for dissecting the molecular mechanisms of neurodegeneration associated with incremental hyperglycaemic stress.

## Introduction

Diabetic retinopathy (DR) is one of the most prevalent complications of diabetes mellitus and stands as a leading cause of vision loss among adults worldwide. The global prevalence of DR was 103 million in 2020 and is expected to reach 160.5 million by 2045 [1]. DR results from chronic hyperglycaemia, which triggers metabolic cascades that progressively damage the retina, leading to visual impairment and blindness. Given the increasing incidence of diabetes globally, DR is a critical focus within both ophthalmologic and systemic diabetic care.

Traditionally, DR has been characterised as a retinal microvascular disorder leading to vascular alterations such as capillary leakage, occlusion, and subsequent ischaemia [2]. However, this vascular-centred view has been recently challenged by accumulating evidence suggesting that retinal neurodegeneration may precede microvascular pathology, contributing to the initiation and progression of DR [3]. Studies indicate that retinal ganglion cells, photoreceptors, and other retinal neurons may undergo early, diabetes-induced degenerative changes, setting the stage for the later vascular complications [4].

Additionally, diabetes mellitus during pregnancy introduces an often-overlooked complication that can impact both the mother and the developing foetus. Maternal hyperglycaemia exposes the foetal retina to elevated glucose levels, potentially disrupting early retinal development and increasing the risk of long-term visual impairment [5, 6]. Despite this, we lack suitable models to study how hyperglycaemia affects the developing retina.

Current DR research relies heavily on animal models to elucidate disease mechanisms and assess potential therapies. However, animal models present limitations, as they often fail to fully replicate human retinal physiology and pathology, especially with respect to the neurodegenerative components of DR. These challenges highlight the need for human-based in vitro models that can accurately recapitulate the complex cellular architecture and functional attributes of the human retina.

Retinal development is a highly conserved process, and human retinal organoids follow a differentiation timeline that mirrors in vivo retinal formation. These organoids generate key retinal cell types, including retinal ganglion cells, photoreceptors, bipolar cells, and Müller glia, in a structured manner. Importantly, they undergo photoreceptor maturation, exhibiting the formation of outer segments (OSs) necessary for phototransduction. Given these attributes, retinal organoids provide a unique platform for investigating the cellular and molecular mechanisms underlying DR.

Previous studies have used retinal organoids to model aspects of DR and hyperglycaemic stress, yet most have relied on extreme glucose concentrations (≥33–75 mM) that exceed physiological relevance and often induce non-specific cytotoxicity [7, 8]. While such systems provide valuable insights into stress and inflammatory responses, they do not capture the progressive nature of hyperglycaemia experienced in patients. Moreover, animal models of DR, such as streptozotocin-induced diabetes in mice or rats, often diverge from human pathology, particularly in the timing and extent of neuronal degeneration and glial activation ([9] and reviewed in [10]). Our work builds upon these studies by modelling incremental hyperglycaemic stress in human retinal organoids, a relevant model that mimics the modest glucose elevations occurring in gestational diabetes or poor glycaemic control in chronic diabetes. This approach allows us to explore early neurodegenerative and glial responses in a human-specific, three-dimensional context that more closely parallels the human retina.

Here, we utilised human retinal organoids as a versatile and scalable model to investigate the effects of incremental hyperglycaemic stress on retinal development and function, simulating scenarios such as gestational diabetes or poor glycaemic control in chronic diabetes, where incremental increases in already elevated glucose may occur and exacerbate retinal stress. Starting from a widely used high-glucose baseline (17.5 mM) necessary for long-term survival and maturation of retinal organoids, we increased glucose levels by 7.5 mM to assess the molecular and cellular consequences in organoids at early and late stages of differentiation. Our findings reveal that early-stage retinal organoids exhibit remarkable resilience to this modest glucose increase, with no significant morphological, viability, or molecular changes. However, as the organoids progress through differentiation, hyperglycaemia induces notable molecular disruptions, including the downregulation of photoreceptor-specific genes, increased apoptosis, and an enhanced oxidative stress response. These changes indicate the vulnerability of photoreceptors to incremental glucose elevation while demonstrating resilience in other retinal cell types, including bipolar cells, ganglion cells, and Müller glia. Our transcriptomic analysis reveals distinct gene expression profiles and enriched pathways disrupted by hyperglycaemia, including phototransduction, sensory perception, and the G protein-coupled receptor signalling pathway. Furthermore, we observed glial activation, marked by upregulation of astrocytic markers, indicative of a reactive gliosis response in hyperglycaemic conditions. Although this non-vascularised retinal organoid system does not capture microvascular pathology or the earliest DR changes from physiological glucose levels, it provides a human-specific platform for elucidating neuronal and glial mechanisms of hyperglycaemic damage and for identifying potential therapeutic targets.

## Materials and methods

### hiPS cell culture

The hiPS cell lines N5 and M8, derived from skin fibroblasts of healthy donors, were used in this study as previously described [11–13]. The cells were maintained in Essential 8 culture medium (Gibco, Thermo Fisher Scientific, Waltham, MA, USA) supplemented with 1× Penicillin–Streptomycin solution (Biosera, Chloet, France) on plates coated with Vitronectin (Thermo Fisher Scientific, Waltham, MA, USA). hiPS cells were cultured at 37 °C in a humidified incubator with 5% CO_2_ to ensure optimal growth conditions. For routine passaging, hiPS cells were dissociated using 0.5 mM EDTA, following standard protocols for gentle handling of pluripotent cells. Cell culture media were refreshed daily, and cells were passaged every 4–5 days. Cell lines were regularly tested for mycoplasma contamination.

### Retinal organoids differentiation

Retinal organoids were generated following previously reported protocols [11–14] with slight modifications. hiPS cells maintained in Essential 8 medium (Gibco) were seeded into U-shaped, cell-repellent 96-well plates (Greiner Bio-One GmbH, Frickenhausen, Germany) at a density of 5000 cells per well. After 48 hours (designated as day 0 of differentiation), the culture medium was replaced with a growth factor-free chemically defined medium (gfCDM). The gfCDM comprised 45% Iscove’s Modified Dulbecco’s Medium (IMDM, Gibco), 45% Ham’s F12 Nutrient Mix (F12, Gibco), 10% KnockOut Serum Replacement (Gibco), 1% chemically defined lipid concentrate (Gibco), 1% Penicillin-Streptomycin Solution (Biosera), and 10 μM β-mercaptoethanol (Sigma-Aldrich, St. Louis, MO, USA). On day 6, recombinant human BMP4 (PeproTech, Cranbury, NJ, USA) was added to the medium at a final concentration of 1.5 nM. Half of the medium volume was replaced with fresh medium every three days. On day 18, the gfCDM was replaced with a neuroretinal (NR) medium composed of DMEM/F12 (Gibco), 1% N-2 supplement (Gibco), 1% GlutaMAX supplement (Gibco), 10% fetal bovine serum (FBS, Biosera), 0.5 mM retinoic acid (Merck KGaA, Darmstadt, Germany), 0.1 mM taurine (Merck), and 1% Penicillin–Streptomycin Solution (Biosera). Organoids were maintained in the 96-well plates until day 18, after which they were transferred to 10 cm Petri dishes for continued culture.

### Glucose treatment

To model incremental hyperglycaemic stress, retinal organoids were cultured in a standard high-glucose baseline and then exposed to a modest glucose increase for 28 days starting at D30, D90, or D150. The baseline medium contained 17.5 mM D-glucose (standard in retinal organoid culture to maintain long-term viability, lamination, and photoreceptor maturation). For the main experiments (all stages unless noted), organoids were assigned to: control–maintenance medium with 17.5 mM D-glucose; D-glucose (hyperglycaemia): baseline medium supplemented with D-glucose (Merck KGaA) to a final 25 mM D-glucose; L-glucose (osmotic control): baseline medium supplemented with L-glucose (Merck KGaA) by 7.5 mM (total glucose equivalents 25 mM). Media were changed twice weekly for 28 days. Additionally, we tested mannitol (7.5 mM; Merck KGaA), added to the baseline 17.5 mM D-glucose medium, to select the most suitable osmotic control. Mannitol and L-glucose were compared for their potential non-specific osmotic effects.

### RNA extraction and reverse transcription quantitative real-time PCR (RT-qPCR)

Retinal organoids were washed twice with PBS and dissociated in RNA Blue Reagent (Top-Bio, Vestec, Czech Republic). Samples were either stored at −80 °C or processed immediately. Organoids were homogenised using an insulin syringe, and total RNA was extracted using the Direct-zol™ RNA MicroPrep Kit (ZYMO Research, Irvine, CA, USA), following the manufacturer’s protocol. Complementary DNA (cDNA) was synthesised from the extracted RNA using the High-Capacity cDNA Reverse Transcription Kit (Applied Biosystems™, Thermo Fisher Scientific). Quantitative PCR was carried out with SYBR Green I (Top-Bio) using the LightCycler® 480 system (Roche Diagnostics GmbH, Mannheim, Germany). Primer sequences are listed in Supplementary Table S3. The expression levels of target genes were normalised against the housekeeping gene GAPDH, and relative fold changes were calculated using the ∆∆Ct method.

### Immunofluorescence staining

Organoids were washed with PBS and fixed in 4% paraformaldehyde (PFA) solution in PBS for 30 minutes at room temperature. After fixation, the PFA was removed, and organoids were washed three times with PBS. They were then incubated overnight in 30% sucrose (Sigma-Aldrich) solution at 4 °C for cryoprotection. Subsequently, organoids were transferred into plastic disposable base moulds, the remaining sucrose solution was carefully removed, and the moulds were filled with Tissue-Tek® O.C.T. Compound medium (Sakura Finetek Europe B.V., Alphen aan den Rijn, The Netherlands). The moulds containing organoids were placed on dry ice for 15 minutes to solidify the medium and stored at −20 °C or processed immediately. Frozen blocks were sectioned into 7 µm-thick slices using a Leica CM1850 Cryostat (Leica Biosystems, Nussloch, Germany). Slides were allowed to dry at room temperature for 20 minutes before further processing. Sections were washed three times with PBS and incubated for 1 hour in blocking buffer (0.3% Triton-X-100, 5% normal goat serum, in PBS) to reduce non-specific binding. The blocking buffer was then removed, and the slides were incubated overnight at 4 °C in a humidified chamber with primary antibodies diluted in antibody diluent (0.3% Triton-X-100, 1% BSA, in PBS). Primary antibodies and their concentrations are listed in Supplementary Table S4. After incubation with primary antibodies, slides were washed three times with antibody diluent (3 minutes per wash) and then incubated with secondary antibodies for 1 hour at room temperature in a humidified chamber. Secondary antibodies included Goat anti-Rabbit IgG (H + L) Alexa594 (1:1000, Thermo Fisher Scientific) and Goat anti-Mouse IgG (H + L) Alexa488 (1:1000, Thermo Fisher Scientific). Cell nuclei were counterstained with DAPI (4′,6-diamidino-2-phenylindole; 1 µl in 1 ml of antibody diluent) for 5 minutes. Slides were then washed five times with PBS, followed by one final rinse in distilled water. The sections were mounted using Fluoroshield™ mounting medium (Sigma-Aldrich), and coverslips were sealed with transparent nail polish to prevent movement or drying. Samples were imaged using a Zeiss LSM 880 laser scanning confocal microscope equipped with the AiryscanFast module (Carl Zeiss Microscopy GmbH, Jena, Germany). Image processing and analysis were performed using the Fiji platform [15]. Quantification of DAPI nuclei and cleaved Caspase-3 cells was performed using QuPath (v0.5.1). Following maximum intensity projection of the merged z-stack images, nuclei were identified based on the DAPI signal. Cell detection was run with the following parameters: radius = 1, sigma = 1.5, minimum and maximum area = 5–500 pixels², and threshold = 400. The resulting cell counts were used for statistical analyses.

### Human apoptosis array

Organoids were washed thoroughly with PBS (10 washes) to remove residual media and lysed in lysis buffer containing 1% SDS, 10% glycerol, and 50 mM TRIS-HCl (pH 6.8). The lysates were sonicated using an ultrasonic homogeniser (Sonopuls HD 2200, Bandelin Electronic GmbH & Co. KG, Berlin, Germany) to ensure thorough cell disruption and protein solubilisation. Protein concentrations in the lysates were quantified and normalised to ensure equal protein loading across samples. The samples were then applied to the Proteome Profiler™ Human Apoptosis Array Kit (R&D Systems, Minneapolis, MN, USA, Cat. No. ARY009), following the manufacturer’s protocol. After completing the assay, membranes were visualised using the ChemiDoc™ Touch Imaging System (Bio-Rad Laboratories, Hercules, CA, USA). The resulting signals were analysed using the Fiji (ImageJ) software’s Protein Array Analyzer plugin, enabling quantitative evaluation of apoptosis-related protein expression.

### Next-generation sequencing (NGS)

RNA integrity was checked on the Fragment Analyzer using RNA Kit 15 nt (Agilent Technologies, Santa Clara, CA, USA). 500 ng of total RNA was used as input for library preparation using QuantSeq 3′ mRNA-Seq FWD with UDI 12 nt Kit (v.2) (Lexogen GmbH, Vienna, Austria) in combination with UMI Second Strand Synthesis Module for QuantSeq FWD. Quality control for library quantity and size distribution was done using QuantiFluor dsDNA System (Promega Corporation, Madison, WI, USA) and High Sensitivity NGS Fragment Analysis Kit (Agilent Technologies). Final library pool was sequenced on NextSeq using High Output Kit v2.5 75 cycles (Illumina Inc., San Diego, CA, USA) in single-end mode, resulting in a minimum of 10 million reads per sample. Quality check of raw single-end fastq reads was carried out by FastQC [16]. The adapters and quality trimming of raw fastq reads were performed using Trimmomatic v0.39 [17] with settings CROP:250 LEADING:3 TRAILING:3 SLIDINGWINDOW:4:5 MINLEN:35. Trimmed RNA-Seq reads were mapped against the human genome (hs38) and Ensembl GRCh38-p10 annotation using STAR v2.7.3a [18] as a splice-aware short read aligner and default parameters except—outFilterMismatchNoverLmax 0.66 and—twopassMode Basic. Quality control after alignment, concerning the number and percentage of uniquely- and multi-mapped reads, rRNA contamination, mapped regions, read coverage distribution, strand specificity, gene biotypes and PCR duplication was performed using several tools, namely RSeQC v4.0.0 [19], Picard toolkit v2.25.6 [20], Qualimap v.2.2.2 [21]. NGS datasets were processed in RStudio using packages: DESeq2 [22], biomaRt [23], Rsubread [24], EnhancedVolcano [25], pheatmap [26], and clusterProfiler [27]. NGS data are accessible at the GEO database: GSE290024.

### Oxidative stress detection

Oxidative stress was assessed using the CellROX™ Green Reagent (Thermo Fisher Scientific) following the manufacturer’s instructions. Briefly, retinal organoids were incubated in maintenance medium containing 5 µM CellROX™ Green for 1 h at 37 °C. The medium was then removed, and organoids were washed five times with PBS to eliminate unbound dye. Organoids were fixed in 4% PFA for 30 min at room temperature and processed for cryosectioning as described in the Immunofluorescence staining section. Sections were counterstained with DAPI (1 µg/ml) and imaged using the Axio Scan.Z1 slide scanner (Carl Zeiss Microscopy GmbH, Jena, Germany). Fluorescence intensities of CellROX™ Green and DAPI were quantified using Fiji (ImageJ) software. As a positive control, organoids were treated with 200 µM hydrogen peroxide (H₂O₂) for 2 h prior to staining and processing.

### Data analysis and statistics

All experiments included at least three independent biological replicates unless stated otherwise. The exact number of biological replicates (*n*) for each experimental group or condition is provided in the corresponding figure or figure legends. For RT-qPCR experiments, each biological replicate was analysed in technical triplicates, with statistics calculated from the mean of the technical replicates. All individual data points are displayed in the figures. Biological replicates represent either individual retinal organoids (for cleaved caspase-3 or CellROX assessments) or batches of retinal organoids (for bulk approaches), each derived from separate differentiation cultures of human induced pluripotent stem cells (hiPS cells). Investigators were not blinded to group allocation during experiments or analysis.

Statistical analyses and data visualisations were performed using RStudio, employing the ggstatsplot [28] and ggplot2 [29]. Data are presented as individual values overlaid on box or violin plots, or as mean ± standard deviation (s.d.) where specified. For experiments involving more than two conditions, one-way ANOVA was used, followed by either Tukey’s post-hoc test (for equal variances) or Games–Howell post-hoc test (for unequal variances). Normality and homogeneity of variance assumptions were assessed before applying parametric tests; if these assumptions were not met, the appropriate alternative post-hoc method was used. All tests were two-sided, and adjustments for multiple comparisons were applied where applicable. Exact *p*-values are reported in the figure panels, and complete ANOVA *F*- and *p*-values are provided in Supplementary Table S5.

## Results

### High-glucose levels do not affect retinal organoids during the early stage of the differentiation process

**Fig. 1:**
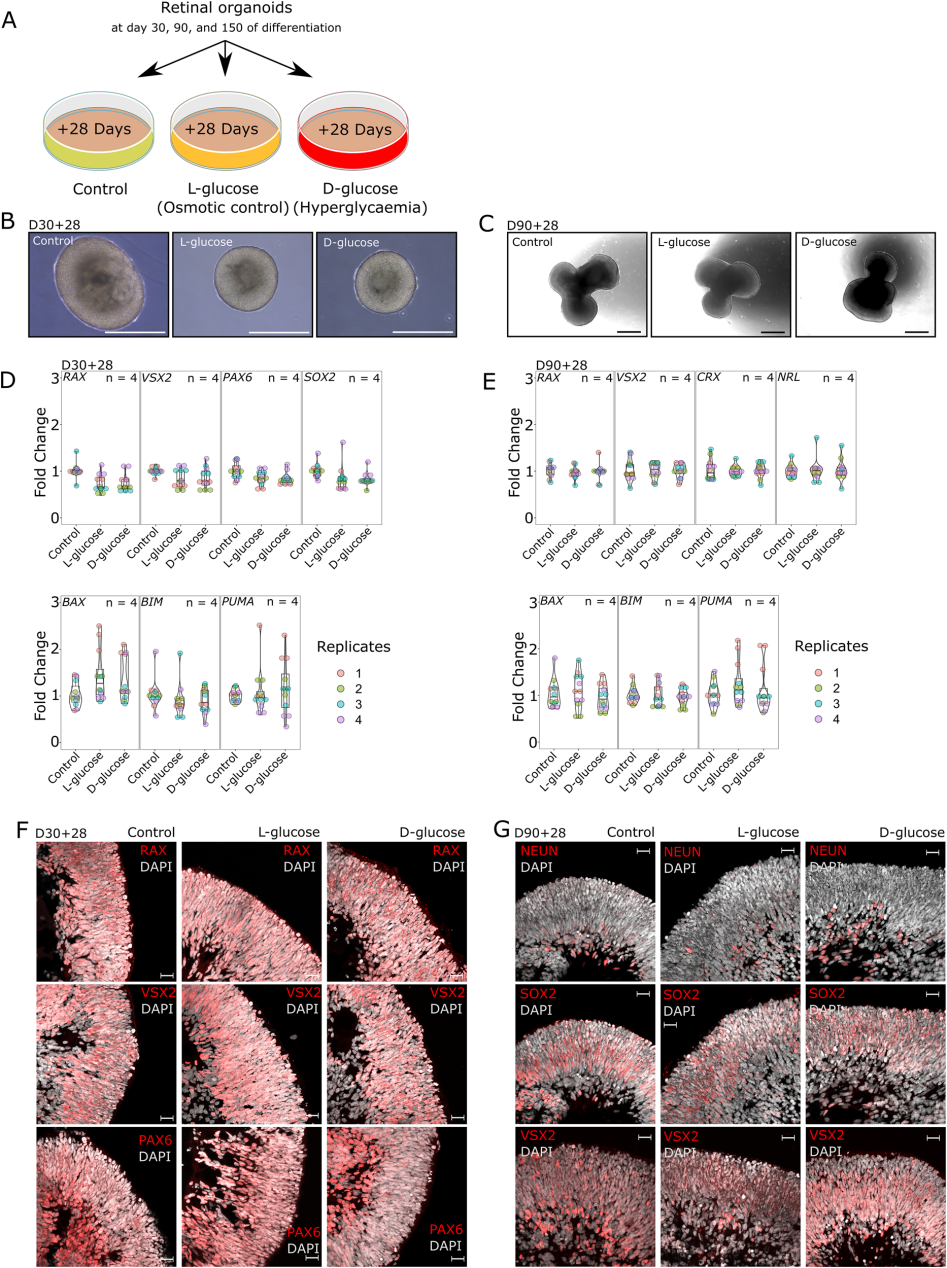
High-glucose levels do not affect retinal organoids during early differentiation. **A** Experimental design of the whole study. Retinal organoids were differentiated for 30, 90, and 150 days (D30, D90, D150) and then treated for 28 days with either Control medium (17.5 mM D-glucose), L-glucose (osmotic control, 17.5 mM D-glucose + 7.5 mM L-glucose), or D-glucose (hyperglycaemia, 25 mM D-glucose). **B** Representative brightfield images of retinal organoids at D30 + 28 for Control, L-glucose, and D-glucose treatment groups, demonstrating similar morphology and no obvious structural abnormalities. Scale bars: 500 µm. **C** Representative brightfield images of retinal organoids at D90 + 28, showing characteristic neuroepithelial morphology across all conditions. Scale bars: 200 µm. **D**, **E** RT-qPCR analysis of gene expression. Results were obtained from retinal organoids derived from two independent cell lines. Individual data points are shown. Statistics: one-way ANOVA. Complete *F*- and *p*-values are provided in Supplementary Table S5. **D** Early retinal and neuronal markers (*RAX, VSX2, PAX6, SOX2*) and apoptosis-related genes (*BAX, BIM, PUMA*) at D30 + 28 and **E** retinal development markers (*RAX, VSX2, CRX, NRL*) and apoptosis-related genes (*BAX, BIM, PUMA*) at D90 + 28 were analysed. **F**, **G** Indirect immunofluorescence staining. **F** Representative images of early retinal markers (RAX, VSX2, PAX6) at D30 + 28. **G** Representative images of retinal and neuronal markers (NEUN, SOX2, VSX2) at D90 + 28. All markers show normal expression and spatial localisation across conditions. DAPI marks nuclei. Scale bars: 20 µm.

We investigated the effects of incremental hyperglycaemic stress on different stages of retinal organoid differentiation. Retinal organoids were differentiated for 30 days, 90 days, and 150 days (D30, D90, D150), followed by 28 days of treatment with either L-glucose (Osmotic control, consisting of the 17.5 mM D-glucose already present in the culture medium plus an additional 7.5 mM L-glucose), D-glucose (Hyperglycaemia, consisting of the 17.5 mM D-glucose already present in the culture medium plus an additional 7.5 mM D-glucose, total 25 mM D-glucose), or the original organoid medium (Control, 17.5 mM D-glucose) (Fig. 1A). It is important to note that while 17.5 mM D-glucose already represents a hyperglycaemic condition compared to physiological levels, this concentration is commonly used in organoid differentiation protocols as a standard baseline to ensure robust development and survival of organoids in vitro [7, 30]. This design, therefore, allowed us to assess the effects of a modest, clinically relevant increase in glucose concentration on tissue already adapted to elevated glucose levels. Additionally, attempts to culture retinal organoids in physiological glucose (5.5 mM D-glucose) resulted in rapid disintegration within seven days (Fig. S1A), confirming the necessity of elevated baseline glucose for long-term survival in vitro.

We first verified the suitability of L-glucose as an osmotic control in this model by comparing it with mannitol, a commonly used osmotic agent in neuronal cultures. Organoids were treated for 28 days with control medium (17.5 mM D-glucose), L-glucose (17.5 + 7.5 mM), or mannitol (17.5 mM D-glucose + 7.5 mM mannitol). While early-stage organoids (D30 + 28) showed no significant changes in pro-apoptotic genes (BAX, BIM, PUMA) among these treatments (Fig. S1B), late-stage organoids (D150 + 28) exposed to mannitol exhibited a trend toward increased BAX expression (*p* = 0.058) and elevated BIM levels, whereas L-glucose had minimal effect (Fig. S1C). These results indicate that mannitol may induce mild apoptotic responses in mature retinal organoids, whereas L-glucose provides a more stable osmotic control and was therefore used in subsequent experiments.

In retinal research and other neuronal culture systems, L-glucose and mannitol are commonly used as osmotic controls to distinguish the metabolic effects of D-glucose from osmotic stress. To evaluate the suitability of these controls in our retinal organoid model, we compared the expression of pro-apoptotic genes *BAX, BIM*, and *PUMA* in organoids treated for 28 days with control medium (17.5 mM D-glucose), medium supplemented with L-glucose (final glucose concentration 25 mM), or medium supplemented with mannitol (7.5 mM). In early-stage organoids (D30 + 28), there were no statistically significant differences in *BAX, BIM*, or *PUMA* expression between the groups (Fig. S1B). In contrast, in late-stage organoids (D150 + 28), mannitol-treated samples showed a trend toward increased *BAX* expression compared with both control and L-glucose (one-way ANOVA, *p* = 0.058) and a marked elevation of *BIM* levels, while *PUMA* remained unchanged (Fig. S1C, Table S5). These data indicate that mannitol may elicit mild pro-apoptotic responses in mature retinal organoids, whereas L-glucose appears to have minimal effect under the same conditions. These findings align with previous reports that, although widely used as an osmotic control, mannitol can induce apoptotic responses in various cell types when applied at higher concentrations, including renal epithelial cells and vascular endothelium [31, 32]. Furthermore, the inclusion of an osmotic control (L-glucose) helps to separate the specific effects of glucose metabolism from osmotic stress. This experimental design ensures that the observed differences are attributable to hyperglycaemia-induced molecular and cellular responses, rather than variability in baseline culture conditions.

Our data indicate that the early stages (D30 + 28, D90 + 28) of retinal organoid differentiation are not significantly affected by hyperglycaemic conditions. Retinal organoids cultured under high-glucose conditions showed no signs of increased cell death and exhibited normal morphologies comparable to control groups. Across all conditions, the organoids successfully formed neuroepithelial structures with characteristic organisation for this stage of differentiation (Fig. 1B, C).

To further investigate the potential impact of hyperglycaemia on early retinal development, we conducted RT-qPCR analysis to quantify the expression levels of retinal and neuronal markers. Specifically, *RAX*, *VSX2*, *PAX6*, and *SOX2* were analysed at D30 + 28, while *RAX, VSX2, CRX*, and *NRL* were analysed at D90 + 28. As shown in Fig. 1D, E, there were no statistically significant changes in the expression levels of these genes among the control, L-glucose (osmotic control), and D-glucose (hyperglycaemia) groups. This suggests that increased glucose levels do not adversely influence the expression of these critical developmental genes. In addition to retinal markers, we assessed the expression levels of apoptosis-related genes (*BAX*, *BIM*, and *PUMA*) using RT-qPCR. Our results showed no significant differences in the expression of these genes across the treatment groups, suggesting that hyperglycaemia does not induce apoptosis in retinal organoids during the early stages of differentiation (Fig. 1D, E).

Finally, we examined the localisation and distribution of key retinal and neuronal transcription factors, including RAX, VSX2, and PAX6 at D30 + 28, and NEUN, SOX2, and VSX2 at D90 + 28 in the retinal organoids. Consistent with our RT-qPCR findings, immunofluorescence analysis revealed no detectable abnormalities in the spatial expression patterns of these markers across the different treatment groups. Representative images in Fig. 1F, G demonstrate that RAX, VSX2, PAX6, NEUN, and SOX2 are expressed within the neuroepithelial structures of the retinal organoids, with no visible differences between control and hyperglycaemic conditions.

These results suggest that increased glucose conditions do not affect the early differentiation, structural development, or cell survival of retinal organoids. The preservation of typical morphology, gene expression levels, apoptosis gene regulation, and protein localisation under hyperglycaemic conditions indicates that early retinal differentiation remains resilient to the metabolic challenges associated with elevated glucose levels.

### High-glucose levels impact photoreceptor gene expression in late-stage retinal organoids

Building on our findings from earlier differentiation stages, where hyperglycaemic conditions did not significantly impact retinal organoid morphology, gene expression, or protein expression and localisation, we next investigated whether these effects persisted or emerged during the later stages of development. At D150 + 28, retinal organoids are at an advanced stage of differentiation, with expected progression of photoreceptor maturation.

**Fig. 2:**
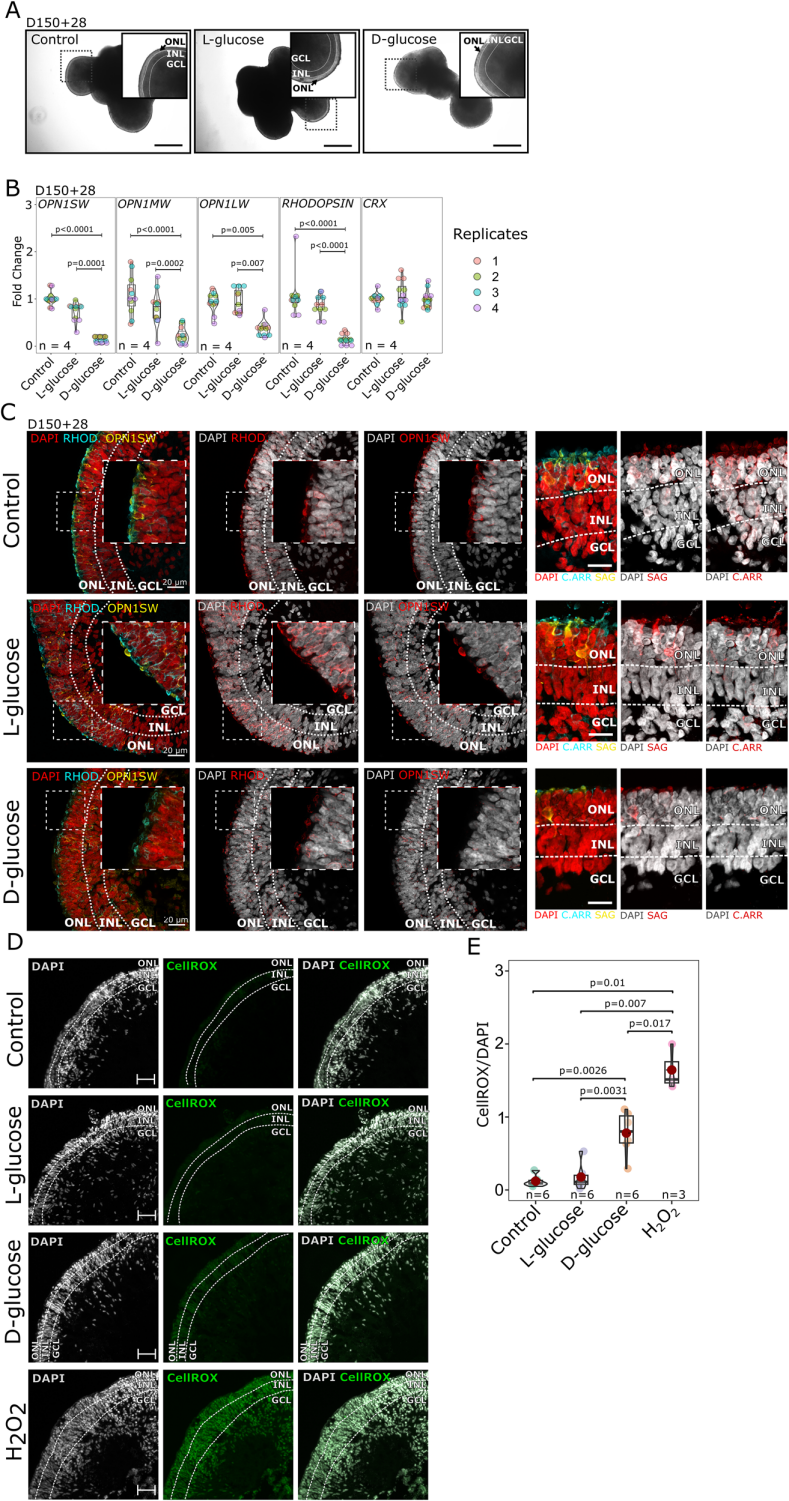
High-glucose levels impact photoreceptor gene expression in late-stage retinal organoids. **A** Brightfield microscopy images of retinal organoids cultured under Control, L-glucose, and D-glucose conditions. Insets highlight enlarged regions of the organoids, with annotated retinal layers: inner nuclear layer (INL), outer nuclear layer (ONL), and ganglion cell layer (GCL). Scale bar: 100 µm. **B** RT-qPCR analysis of photoreceptor-specific genes *OPN1SW, OPN1MW, OPN1LW*, and *RHODOPSIN* shows significant downregulation in the D-glucose group compared to Control and L-glucose groups. *CRX* expression remains unchanged across all conditions. Results were obtained from retinal organoids derived from two independent cell lines. Individual data points are shown. Statistics: one-way ANOVA followed by Tukey’s post-hoc test. Complete *F*- and *p*-values are provided in Supplementary Table S5. **C** Immunofluorescence staining of retinal organoids for photoreceptor markers. Left panel: OPN1SW and RHODOPSIN staining reveal reduced expression in the D-glucose group compared to the Control and L-glucose groups. Right panel: C.ARR (Cone arrestin, ARR3) and SAG (Rod arrestin) also exhibit reduced expression in the D-glucose group. DAPI marks nuclei. Insets show magnified regions of interest. Scale bars: 20 µm. ONL outer nuclear layer, INL inner nuclear layer, GCL ganglion cell layer. **D** Detection of reactive oxygen species (ROS) in retinal organoids using the CellROX™ Green reagent. Treatment with 200 µM hydrogen peroxide (H₂O₂) for 2 hours served as a positive control and showed a robust CellROX signal throughout the tissue. Scale bars: 50 µm. ONL outer nuclear layer, INL = inner nuclear layer, GCL ganglion cell. **E** Quantification of CellROX fluorescence intensity normalised to DAPI signal. Individual values are shown (*n* = 6 for Control, L-glucose, and D-glucose; *n* = 3 for H₂O₂), with each n representing one organoid. Statistical analysis was performed using one-way ANOVA followed by Games–Howell post-hoc test; exact *p*-values are indicated on the plot. Complete ANOVA F- and *p*-values are provided in Supplementary Table S5.

Brightfield microscopy images of the organoids (Fig. 2A) revealed no morphological differences across the treatment groups (Control, L-glucose, and D-glucose), like the observations in earlier stages of differentiation. However, in contrast to the earlier stages, significant differences in key retinal-specific gene expression emerged at D150 + 28.

RT-qPCR analysis was performed to measure the expression levels of photoreceptor-specific genes, including *OPN1SW* (short-wavelength-sensitive opsin), *OPN1MW* (medium-wavelength-sensitive opsin), *OPN1LW* (long-wavelength-sensitive opsin), and *RHODOPSIN*. The results showed a significant downregulation of expression of all four genes in the D-glucose group compared to the controls (Fig. 2B), suggesting that D-glucose treatment negatively impacts the expression of critical photoreceptor markers. Interestingly, *CRX*, a key transcription factor involved in photoreceptor development, remained unchanged across all conditions (Fig. 2B).

Immunofluorescence staining revealed reduced OS structures and expression of photoreceptor markers in the D-glucose group compared to the control and L-glucose groups (Fig. 2C). Both OPN1SW and RHODOPSIN showed decreased levels, consistent with the RT-qPCR results. Additionally, cone arrestin (C.ARR, ARR3) and SAG (S-antigen or arrestin-1) were also downregulated, indicating a broader disruption in photoreceptor differentiation under D-glucose conditions.

To further assess oxidative stress under hyperglycaemic conditions, we employed the CellROX™ reagent to directly visualise reactive oxygen species (ROS) in retinal organoids. Minimal or no ROS signal was observed in control and L-glucose-treated organoids, whereas D-glucose treatment led to a pronounced increase in ROS fluorescence, particularly in outer and inner nuclear layers (Fig. 2D, E). As a positive control, organoids exposed to 200 µM hydrogen peroxide (H₂O₂) for 2 hours displayed a strong and widespread CellROX signal. These findings indicate that D-glucose induces a detectable increase in oxidative stress within the organoid tissue.

**Fig. 3:**
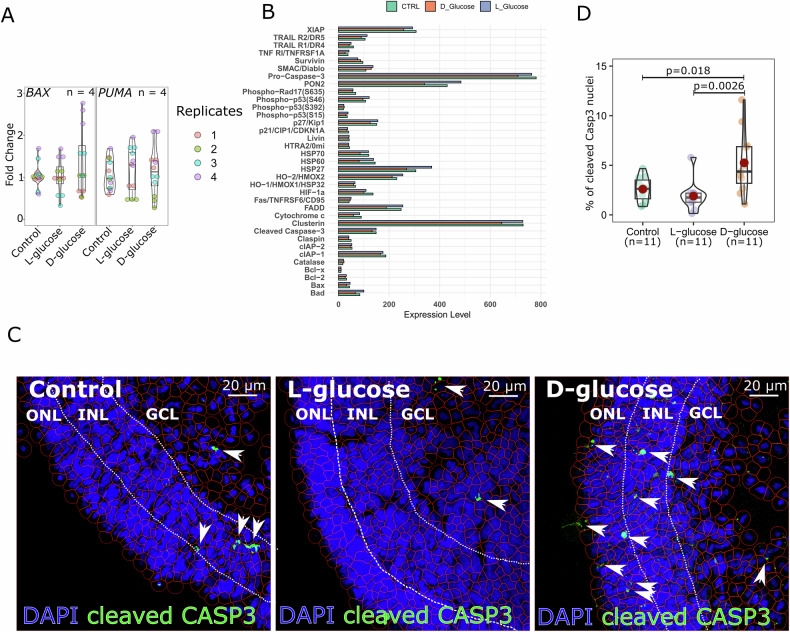
Analysis of apoptosis-related responses in late-stage retinal organoids exposed to hyperglycaemia. **A** RT-qPCR analysis of pro-apoptotic genes (*BAX* and *PUMA*) in retinal organoids after D-glucose, L-glucose, or control treatment at D150 + 28. No significant changes in expression levels were observed across conditions. Results were obtained from retinal organoids derived from two independent cell lines. Individual data points are shown. **B** Quantification of apoptosis-related proteins using a protein array. **C** Representative immunofluorescence images of late-stage (D150 + 28) retinal organoid cryosections stained for cell nuclei (DAPI, blue) and cleaved caspase-3 (green) to detect apoptotic cells. Arrowheads indicate cleaved caspase-3-positive cells. Cell segmentation outlines (red) were generated and quantified using QuPath software. ONL outer nuclear layer, INL inner nuclear layer, GCL ganglion cell layer. Scale bars: 20 µm. **D** Quantification of cleaved caspase-3-positive cells in retinal organoids. Individual values are shown (*n* = 11 for all conditions), with each n representing one organoid. Statistics: one-way ANOVA followed by Tukey’s post-hoc test. Complete *F*- and *p*-values are provided in Supplementary Table S5.

Having observed the downregulation of photoreceptor-specific genes in response to hyperglycaemia, we next investigated whether this might be associated with apoptosis, particularly in photoreceptors. We performed RT-qPCR analysis of key pro-apoptotic genes, including *BAX* and *PUMA* (Fig. 3A). However, no significant differences were observed between the control, L-glucose, and D-glucose groups, suggesting that the transcriptional regulation of apoptosis-related genes remains unchanged across conditions. Additionally, we performed a more comprehensive analysis of apoptosis-related proteins using protein arrays (Figs. 3B and S2). This analysis also revealed no global upregulation or activation of apoptosis-related markers in bulk protein arrays.

Recognising that bulk approaches may obscure cell-specific responses, we turned to immunofluorescence staining for cleaved caspase-3, a marker of apoptosis, to localise apoptotic activity within the organoids (Fig. 3C). Quantification of cleaved caspase-3-positive cells using cell segmentation (Fig. 3D) revealed a statistically significant increase in apoptotic cells in the D-glucose group compared to both control and L-glucose groups. The apoptotic cells were primarily localised within the outer nuclear layer, suggesting that late-stage photoreceptors are particularly vulnerable to even a modest (7.5 mM) increase in glucose above baseline culture conditions.

### Transcriptomic analysis revealed profound alterations in photoreceptor-specific gene expression

**Fig. 4:**
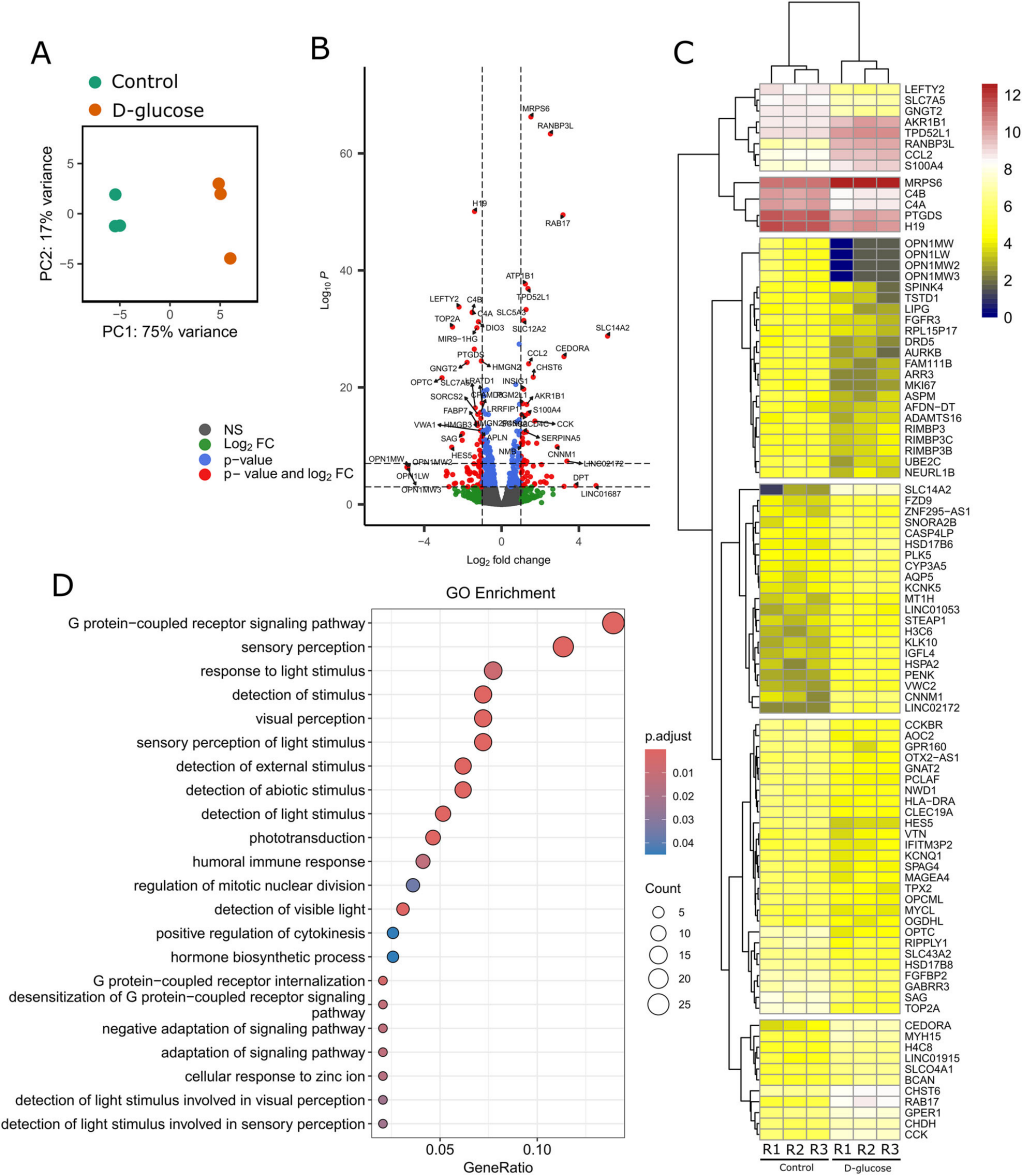
Transcriptomic analysis of retinal organoids exposed to hyperglycaemia. **A** Principal component analysis (PCA) plot showing the clustering of transcriptomic profiles. Control and D-glucose-treated groups form distinct clusters. **B** Volcano plot illustrating differential gene expression between retinal organoids treated with D-glucose and controls. Significantly upregulated and downregulated genes are highlighted based on adjusted *p*-value and log2 fold change. **C** Heatmap displaying expression patterns of differentially expressed genes. **D** Gene Ontology (GO) enrichment analysis of all differentially expressed genes (*p* < 0.05, log2FoldChange ≥1).

To gain a comprehensive understanding of the molecular changes induced by hyperglycaemia, we performed RNA sequencing on retinal organoids treated with D-glucose (D150 + 28) compared to controls. Principal component analysis revealed distinct transcriptomic profiles between the control and D-glucose-treated organoids (Fig. 4A). Control replicates formed a tightly clustered group, while D-glucose replicates grouped separately, indicating that hyperglycaemic treatment induces widespread and reproducible changes in gene expression patterns within retinal organoids.

We found 251 differentially expressed genes (*p* < 0.05, log2FoldChange ≥ 1) (Supplementary Table S1). Among the downregulated genes, *OPN1MW*, *OPN1LW*, *ARR3* and *SAG* stood out as key photoreceptor-specific genes that exhibited significantly reduced expression under hyperglycaemic conditions (Fig. 4B, C). These genes are critical for cone and rod photoreceptor function, and their downregulation highlights the vulnerability of photoreceptor differentiation and maturation to elevated glucose levels. Gene Ontology (GO) enrichment analysis (Fig. 4D, S3) revealed that the most significantly enriched pathways were G protein-coupled receptor signalling pathway, sensory perception, response to light stimulus, and visual perception.

To evaluate the consistency and complementarity of our findings, we conducted a comparative analysis between our NGS dataset and the publicly available dataset GSE160306. The GSE160306 dataset consists of transcriptomic profiles from human retinal samples, including control and DR conditions, enabling a comprehensive exploration of gene expression changes associated with hyperglycaemia and DR [33]. We identified 50 overlapping differentially expressed genes (*p* < 0.05) between our dataset and GSE160306 (Fig. S4A, Supplementary Table S2). GO analysis of these shared genes revealed that they play a role in key biological processes, including gliogenesis, eye development, and visual and sensory system development (Fig. S4B). Next, both datasets were subjected to GO analysis to elucidate enriched biological processes and pathways. Our primary focus was to assess how the dataset GSE160306 integrates into the pathways identified in our GO analysis, providing a comprehensive view of shared and distinct mechanisms. Consistent with our datasets, we found significant overlaps in pathways related to retina, including eye development, visual and sensory system development, gliogenesis, and glial cell differentiation (Fig. S4C). One of the notable findings from integrating GSE160306 into our dataset was the strong emphasis on photoreceptor-related gene expression changes under hyperglycaemic conditions. This aligned with our observations of altered photoreceptor-related pathways in hyperglycaemic organoids.

### Hyperglycaemia increases the oxidative stress response

**Fig. 5:**
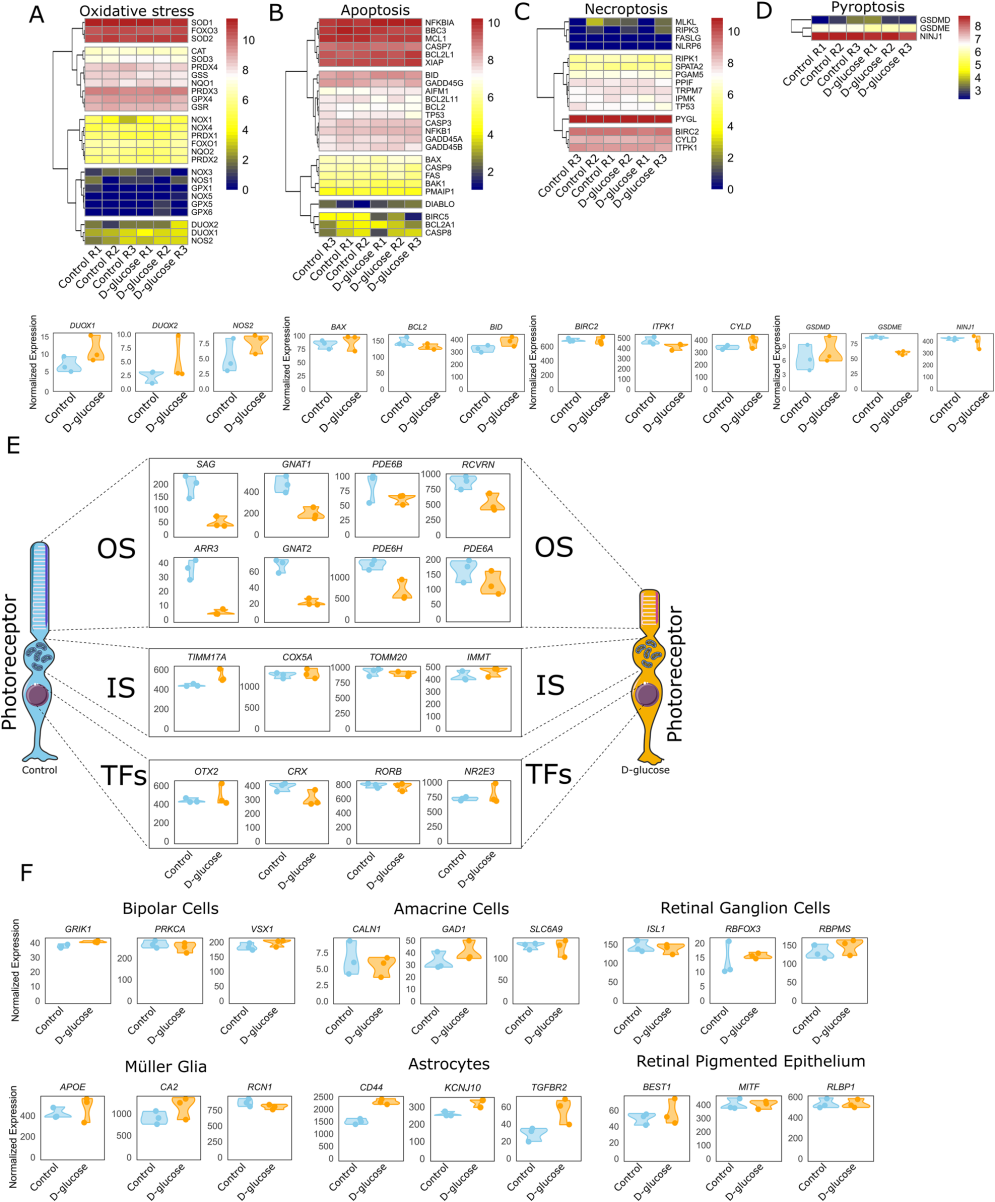
Gene expression analysis of oxidative stress, apoptosis, necroptosis, pyroptosis, and cell-specific markers in retinal organoids under incremental hyperglycaemic stress. **A**–**D** Heatmap and violin plots showing the expression patterns of: **A** oxidative stress-related genes in retinal organoids treated with D-glucose compared to controls. Key genes include *DUOX1, DUOX2*, and *NOS2*. **B** apoptosis-related genes, including *BAX, BCL2*, and *BID*. C) necroptotic process. Key genes: *BIRC2, ITPK1*, and *CYLD*. **D** pyroptotic cell death. Key genes: *GSDMD, GSDME*, and *NINJ1*. **E** Expression of photoreceptor-specific genes; outer segment (OS) (rod-specific: *SAG, GNAT1, PDE6B, RCVRN*; cone-specific: *ARR3, GNAT2, PDE6H, PDE6A*); inner segment (IS) (*TIMM17A, COX5A, TOMM20, IMMT*); photoreceptor-specific transcription factors (TFs) (*OTX2, CRX, RORB, NR2E3*), **F** Expression of cell-specific markers across different retinal cell types, including bipolar cells (*GRIK1, VSX1, PRKCA*), amacrine cells (*CALN1, GAD1, SLC6A9*), retinal ganglion cells (*ISL1, RBFOX3, RBPMS*), Müller glia (*APOE, CA2, RCN1*), astrocytes (*CD44, KCNJ10, TGFBR2*), and retinal pigmented epithelium (*BEST1, MITF, RLBP1*). The data represent individual data points across three biological replicates for each condition.

To further investigate the molecular impacts of hyperglycaemia on retinal organoids, we analysed gene expression related to oxidative stress, apoptosis, necroptotic process, pyroptotic cell death, and cell-specific markers for various retinal cell types. Genes associated with oxidative stress regulation showed differential expression patterns. Notably, *DUOX1* and *DUOX2* were upregulated in the D-glucose condition (Figs. 5A and S5A), indicating an active response to elevated oxidative stress. In contrast, other key oxidative stress-related genes, including *CAT, SOD1, SOD2*, and *NOX1, NOX3*, and *NOX4*, displayed no profound changes. Interestingly, although *SOD2* expression was not upregulated at the transcript level in our NGS dataset, immunostaining revealed a clear increase in SOD2 protein levels in the D-glucose condition (Fig. S5B), suggesting post-transcriptional regulation or protein stabilisation under hyperglycaemic stress. These findings suggest a complex, context-dependent oxidative stress response in retinal organoids under hyperglycaemic conditions, wherein some pathways are activated while others remain unaffected. In the apoptosis-related gene set (Fig. 5B), no pro-apoptotic genes were upregulated in response to D-glucose. However, the anti-apoptotic gene *BIRC5* (survivin) was downregulated, indicating a subtle shift in the balance of apoptotic regulation. Despite this, the overall apoptotic gene expression profile reflects a tightly regulated response, potentially preventing an overt induction of apoptosis under hyperglycaemic stress. Necroptosis markers (Fig. 5C) and pyroptosis markers (Fig. 5D) were not upregulated in the D-glucose condition, indicating that elevated glucose levels do not induce these forms of programmed cell death in retinal organoids. In addition, immunostaining for RIPK3 (a necroptosis marker) and GSDMD (a pyroptosis marker) showed no increased expression in the D-glucose condition, further supporting the absence of necroptotic and pyroptotic activation under hyperglycaemic stress (Fig. S5C, D).

Cell-type-specific gene expression analysis (Fig. 5E, F) revealed differential responses among retinal cell markers. Cone and rod photoreceptor OS markers, including *SAG, GNAT1, PDE6B, RCVRN, ARR3, GNAT2, PDE6H*, and *PDE6A*, were markedly downregulated under hyperglycaemic conditions, aligning with previous findings of impaired photoreceptor-specific gene expression. Interestingly, inner segment (IS) markers, such as *TIMM17A, COX5A, TOMM20*, and *IMMT*, along with photoreceptor-specific transcription factors including *OTX2, CRX, NR2E3*, and *RORB*, remained largely unaffected. This suggests that hyperglycaemia primarily disrupts downstream photoreceptor differentiation and functional maturation, rather than early developmental programming. The selective vulnerability of OS-associated genes suggests that photoreceptor OSs are particularly sensitive to glucose-induced stress, potentially contributing to progressive photoreceptor dysfunction in DR.

In contrast, markers for bipolar cells (*GRIK1, VSX1, PRKCA*), amacrine cells (*CALN1, GAD1, SLC6A9*), retinal ganglion cells (*ISL1, RBFOX3, RBPMS*), Müller glia (*APOE, CA2, RCN1*), and retinal pigment epithelial cells (*BEST1, MITF, RLBP1*) remained largely unchanged. These findings highlight a notable resilience of these retinal cell types to hyperglycaemic stress, suggesting that photoreceptors are particularly vulnerable to glucose-induced damage. Interestingly, astrocyte markers (*CD44*, *KCNJ10*, and *TGFBR2*) were upregulated under hyperglycaemia in the NGS dataset. Consistent with these transcriptional changes, immunostaining confirmed increased expression of the astrocytic proteins GFAP and S100β in D-glucose-treated organoids compared to controls (Fig. S5E). Closer examination of GFAP staining revealed a pronounced extension of GFAP-positive processes, indicative of morphological activation typically associated with reactive gliosis. Together, these findings suggest an increased gliotic response or astrocyte activation, which may represent a compensatory mechanism to counteract hyperglycaemia-induced stress in the retinal environment.

## Discussion

In this study, we utilised human retinal organoids as a model to investigate the impact of hyperglycaemia on retinal development across various stages of differentiation. Our results demonstrate that while early-stage retinal organoids exhibit resilience to hyperglycaemic conditions, prolonged exposure at later stages induces significant molecular and cellular disruptions, particularly in photoreceptor differentiation and oxidative stress responses.

In the early stages of differentiation (D30 + 28 and D90 + 28), retinal organoids treated with hyperglycaemic conditions showed no significant differences in morphology or structure compared to controls. These findings suggest that the early differentiation processes, such as neural induction and retinal progenitor specification, remain unaffected by hyperglycaemic stress. One possible explanation is that early-stage retinal cells have distinct metabolic demands. Given their high metabolic rate, photoreceptor progenitors and other early-stage retinal cells may possess an intrinsic ability to withstand hyperglycaemic conditions more effectively than their mature counterparts [34, 35]. Supporting this idea, in vitro studies have shown that photoreceptor progenitors do not exhibit significant loss under high-glucose exposure, indicating that metabolic requirements could play a key role in their survival [7]. This metabolic resilience may also indicate that the detrimental effects of hyperglycaemia become more pronounced as organoids undergo further differentiation, paralleling the progressive nature of DR observed in vivo. However, as organoids progressed to later stages of differentiation (D150 + 28), hyperglycaemic conditions induced significant transcriptional changes, particularly in photoreceptor-specific genes. Genes such as *OPN1SW, OPN1MW, OPN1LW*, and *RHODOPSIN* (Fig. 2B), along with additional markers (Fig. 5E), were markedly downregulated, highlighting the vulnerability of both cone and rod photoreceptors to prolonged glucose exposure. Strikingly, these effects were elicited by an increment of only 7.5 mM D-glucose above baseline culture conditions—an increase that, while small in absolute terms, was sufficient to trigger significant oxidative stress, transcriptional dysregulation, and apoptotic cell loss in late-stage organoids. This progressive impairment mirrors the pathophysiology of DR, where metabolic dysregulation increasingly affects photoreceptors over time [10]. While bulk gene and protein analyses did not indicate widespread apoptotic activation, quantification of cleaved caspase-3 staining revealed a significant increase in apoptotic cells in D-glucose-treated organoids, localised primarily to the outer nuclear layer. This effect was detected despite the modest glucose increment (from 17.5 to 25 mM), highlighting the pronounced sensitivity of mature photoreceptors to even small elevations in glucose above baseline. These findings extend previous organoid and in vivo studies of DR. For example, de Lemos et al. (2024) [7] reported widespread oxidative and inflammatory changes in retinal organoids exposed to supraphysiologic glucose concentrations (50–75 mM), while Inagaki et al. [8] demonstrated vascular alterations in vascularised retinal organoids cultured under 33 mM glucose. In contrast, our incremental model, raising glucose from 17.5 mM to 25 mM, reveals that even modest increases can elicit oxidative stress, astrocyte activation, and selective photoreceptor vulnerability. This incremental model shows how the magnitude and duration of hyperglycaemic exposure critically shape retinal outcomes and supports the concept that photoreceptor dysfunction may arise before overt vascular pathology, as proposed in clinical and animal studies [10, 34].

Notably, transcription factors critical for photoreceptor development, such as *CRX* and *NRL*, remained unchanged, suggesting that hyperglycaemia selectively impairs photoreceptor terminal differentiation and function rather than their initial specification. This aligns with the concept that mature photoreceptors, which rely heavily on oxidative metabolism, are particularly susceptible to prolonged metabolic stress, further supporting the parallels between glucose-induced transcriptional changes in organoids and photoreceptor degeneration in DR [10]. These findings are consistent with the zebrafish model study by Titialii-Torres et al. [5], which demonstrated photoreceptor vulnerability under hyperglycaemic conditions, characterised by shortened OS. While our model similarly revealed significant downregulation of OS-specific photoreceptor genes, the transcriptomic resolution provided by human retinal organoids offered additional insights, highlighting disruptions in phototransduction pathways and the downregulation of genes whose products are critical for OS function and structure. Interestingly, our datasets emphasise oxidative stress as a central driver of hyperglycaemic damage. The observed upregulation of *DUOX1* and *DUOX2* aligns with the findings of increased ROS production [36, 37], further corroborating the conserved role of oxidative stress in photoreceptor dysfunction. Importantly, this transcriptional upregulation was accompanied by direct functional evidence of oxidative stress, as shown by CellROX™ Green staining, which revealed a pronounced ROS increase in D-glucose-treated organoids compared to both control and L-glucose conditions (Fig. 2D, E). This indicates that the oxidative stress response observed at the gene expression level translates into measurable changes in cellular redox status. While some antioxidant genes did not show significant transcriptional changes, the magnitude of ROS elevation suggests that baseline antioxidant defences are insufficient to fully counteract hyperglycaemia-induced oxidative stress in late-stage organoids.

Cell-type-specific gene expression analysis revealed differential resilience across retinal cell types. While OS photoreceptor markers were downregulated, markers for bipolar cells, amacrine cells, ganglion cells, Müller glia, and retinal pigment epithelial cells remained largely unaffected. This indicates the cell-type-specific resilience of retinal organoids to hyperglycaemic stress. However, astrocyte markers, including *CD44, KCNJ10, TGFBR2*, GFAP, and S100β, were upregulated under hyperglycaemia, as shown by both NGS transcriptomic analysis and immunostaining. This provides converging evidence for astrocyte activation and gliotic remodelling in late-stage organoids. Such activation may arise either as a secondary response to hyperglycaemia-induced neuronal injury or from direct metabolic stress to astrocytes themselves, both of which have been reported in hyperglycaemic retinal tissue. Both our data and the findings of Titialii-Torres et al. [5] support this interpretation, indicating that reactive gliosis is a common early response to retinal metabolic stress that can contribute to both neuroprotection and inflammation.

Our findings demonstrate that retinal organoids effectively model the effects of hyperglycaemia on retinal development, revealing significant oxidative stress responses, photoreceptor vulnerability, and glial activation. These results align with and expand upon the study by de Lemos et al. [7]. Consistent with their observations, we detected significant oxidative stress responses in retinal organoids exposed to hyperglycaemia. While their study identified retinal ganglion cells and amacrine cells as early targets of hyperglycaemia, our analysis revealed resilience in markers for these cell types. This could be explained by differences in experimental design, as their study employed much higher concentrations of D-glucose (50 and 75 mM), levels that far exceed physiological conditions and approach cytotoxicity. Such extreme hyperglycaemia likely triggered more severe stress responses, overwhelming cellular defences and leading to widespread damage. This heightened stress response was associated with increased glial reactivity, marked by elevated *GFAP* expression. Our data similarly showed upregulation of *GFAP* and *CD44*, indicating reactive gliosis in response to hyperglycaemia. This glial activation likely represents a common early response, contributing to both neuroprotection and inflammation in the hyperglycaemic retinal environment.

One important limitation of our approach is that human retinal organoids cannot be maintained long-term at physiological glucose concentrations (~5.5 mM) without rapid structural disintegration (Fig. S1A). This necessitates the use of an elevated baseline glucose concentration (17.5 mM) in differentiation protocols, as reported previously [7, 30]. Consequently, our experimental design does not model the initial transition from normoglycaemia to hyperglycaemia, but rather examines the effects of incremental hyperglycaemic stress on retinal tissue already adapted to a higher glucose environment. Despite this, we found that even a modest increase of 7.5 mM D-glucose (from 17.5 to 25 mM) elicited significant and reproducible changes in photoreceptor gene expression, oxidative stress, astrocyte activation, and apoptosis in late-stage organoids. This demonstrates that retinal organoids remain sensitive to further glucose elevation and can effectively model neurodegenerative processes relevant to scenarios such as gestational diabetes or poor glycaemic control in chronic diabetes. However, retinal organoids might not replicate the full spectrum of chronic DR pathogenesis, as features such as vascular alterations, immune cell interactions, and systemic metabolic influences are absent from this in vitro system. Furthermore, while the absolute glucose concentrations used here are higher than those defining diabetes in vivo (~11.1 mM), they reflect a necessary adaptation for sustaining long-term retinal organoid survival and maturation in vitro. Our design, therefore, models the impact of a relative increase in glucose on tissue already adapted to high-glucose culture.

Comparison of our datasets with publicly available datasets [33] further validated our findings, despite the inherent biological differences between the models. Our data, derived from retinal organoids, lack key components such as immune cells, vasculature, and systemic influences, which are present in human retinal samples. Nevertheless, both datasets revealed significant overlaps in pathways related to retinal development, gliogenesis, and photoreceptor dysfunction. However, there were some differences in the extent to which certain pathways were enriched. For instance, inflammatory and angiogenic processes were more pronounced in the human retinal dataset, likely due to the presence of vascular and immune components. These differences likely contribute to the distinct pathway representations observed between models. While retinal organoid models effectively capture DR-associated neurodegenerative processes, in vivo systems provide essential insights into the vascular and immune components that drive disease progression. Ongoing efforts to generate vascularised retinal organoids or model the blood-retinal barrier may help bridge this gap, allowing for a more accurate representation of in vivo pathophysiological mechanisms and enhancing the translational potential of these models [38]. In non-vascular systems such as ours, nutrient and oxygen supply rely solely on passive diffusion, which may limit penetration into the inner retinal layers. This limitation is particularly relevant given that DR is known to predominantly cause cell death in the inner retinal layers [9], meaning that such pathology may be underrepresented in our current model. Incorporating vascular networks could facilitate nutrient exchange, improve metabolic support for inner retinal neurons, and enable modelling of cell types and degenerative processes that are less accessible in current non-vascularised organoid models [8]. Despite this, the model is important as it revealed a neurotoxic effect of hyperglycaemia, highlighting specific pathways and molecular mechanisms underlying DR that can be further explored in translational research.

Retinal organoids represent a highly effective model for studying the harmful effects of hyperglycaemia on the retina, capturing key features such as oxidative stress responses, photoreceptor vulnerability, and glial activation. Given their close resemblance to human retinal development [39–41], they provide a powerful platform for investigating how maternal hyperglycaemia impacts foetal retinal formation and its long-term consequences. Furthermore, this model enables the development and testing of potential therapeutic strategies to mitigate hyperglycaemia-induced retinal damage, including antioxidant treatments, metabolic modulators, and neuroprotective interventions.

## Supplementary information

Supplementary Figures

Legends for Supplementary Tables

Supplementary Table 1

Supplementary Table 2

Supplementary Table 3

Supplementary Table 4

Supplementary Table 5

## Data Availability

The transcriptomic dataset is available through GEO under accession number GSE290024. Any additional requests for information can be directed to and will be fulfilled by the corresponding author.
