# Supplementary material for: Unveiling the cellular and molecular mechanisms of diabetic retinopathy with human retinal organoids
Source: Cell Death Dis. 2025 Dec 19;16(1):892. doi: 10.1038/s41419-025-08244-1 (PMC12717054; doi:10.1038/s41419-025-08244-1)
Supplement: Supplementary file 1 — Supplementary Figures [file 41419_2025_8244_MOESM1_ESM.pdf]

Supplementary Figures

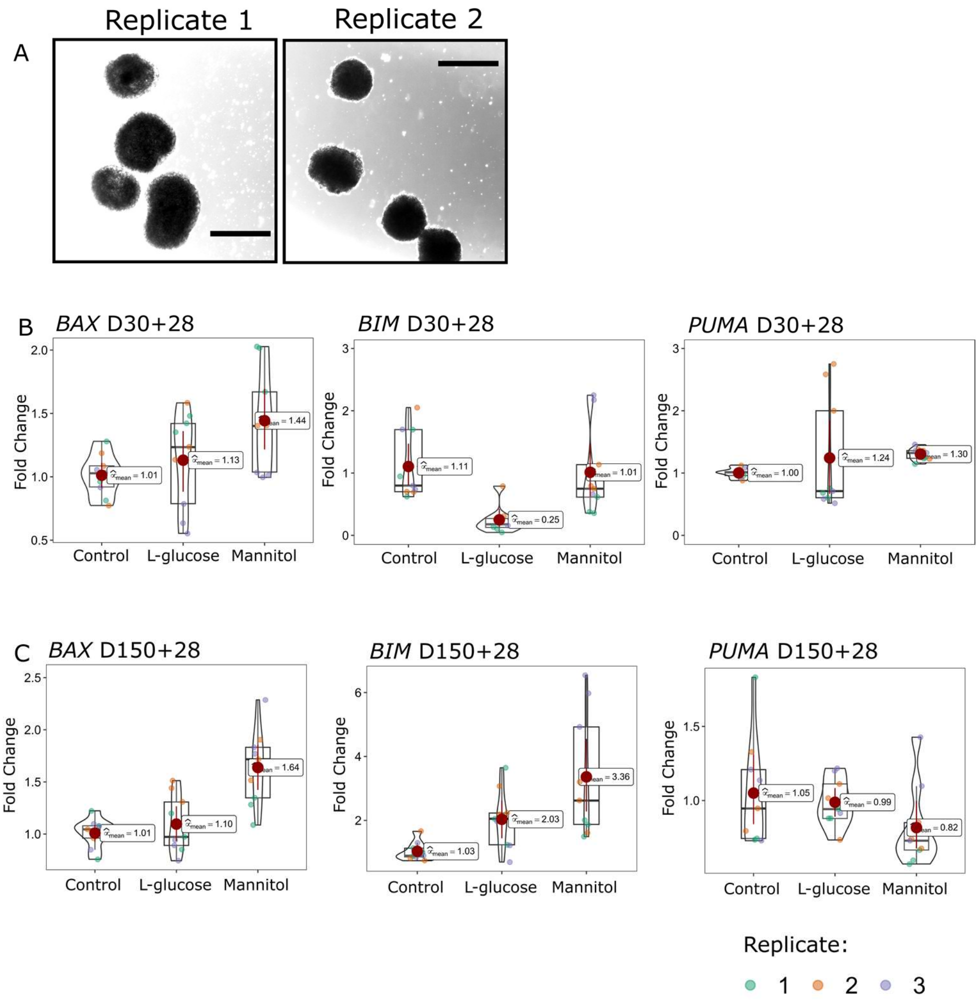

Figure S1: A) Images of retinal organoids at D30 cultured for additional 7 days in 5.5 mM D-glucose. We observed increased cell death and organoid degradation. Scale bars represents 500 µm. **B)** Expression of apoptosis-related genes (*BAX, BIM, PUMA*) in early-stage retinal organoids (D30+28) treated for 28 days with control medium (17.5 mM D-glucose), L-glucose (17.5 mM D-glucose + 7.5 mM L-glucose), or mannitol (17.5 mM D-glucose + 7.5 mM mannitol). Statistics: one-way ANOVA. Complete F- and p-values are provided in Supplementary Table S5. **C)** Expression of the same apoptosis-related genes in late-stage retinal organoids (D150+28) following the same treatments. Mannitol-treated organoids showed a trend toward increased *BAX* expression compared with control and L-glucose and an elevation of *BIM*, whereas *PUMA* remained unchanged. Data are presented from three independent biological replicates. Statistics: one-way ANOVA. Complete F- and p-values are provided in Supplementary Table S5.

A

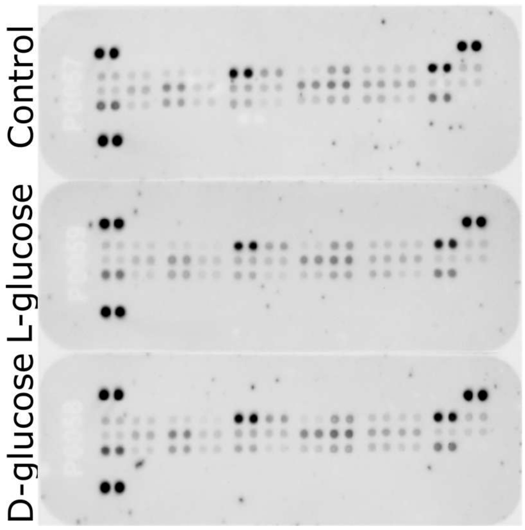

B

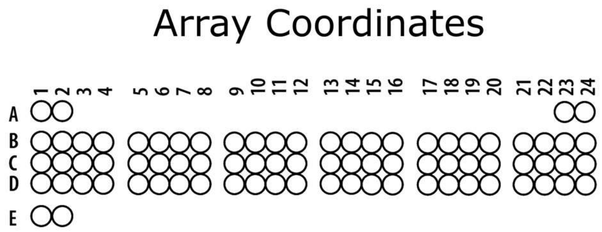

C

Coordinate	Target/Control
A1, A2	Reference Spots
A23, A24	Reference Spots
B1, B2	Bad
B3, B4	Bax
B5, B6	Bcl-2
B7, B8	Bcl-x
B9, B10	Pro-Caspase-3
B11, B12	Cleaved Caspase-3
B13, B14	Catalase
B15, B16	cIAP-1
B17, B18	cIAP-2
B19, B20	Claspin
B21, B22	Clusterin
B23, B24	Cytochrome c
C1, C2	TRAIL R1/DR4
C3, C4	TRAIL R2/DR5
C5, C6	FADD
C7, C8	Fas/TNFRSF6/CD95
C9, C10	HIF-1α
C11, C12	HO-1/HMOX1/HSP32
C13, C14	HO-2/HMOX2
C15, C16	HSP27
C17, C18	HSP60
C19, C20	HSP70
C21, C22	HTRA2/Omi
C23, C24	Livin
D1, D2	PON2
D3, D4	p21/CIP1/CDKN1A
D5, D6	p27/Kip1
D7, D8	Phospho-p53 (S15)
D9, D10	Phospho-p53 (S46)
D11, D12	Phospho-p53 (S392)
D13, D14	Phospho-Rad17 (S635)
D15, D16	SMAC/Diablo
D17, D18	Survivin
D19, D20	TNF RI/TNFRSF1A
D21, D22	XIAP
D23, D24	PBS (Negative Control)
E1, E2	Reference Spots

Figure S2: A) Images of protein arrays used to analyse apoptosis-related markers in retinal organoids exposed to hyperglycaemic conditions. **B)** Corresponding coordinates on the chip indicating the placement of individual protein spots. **C)** A reference table listing the coordinates and the respective protein symbols for the detected apoptosis-related markers.

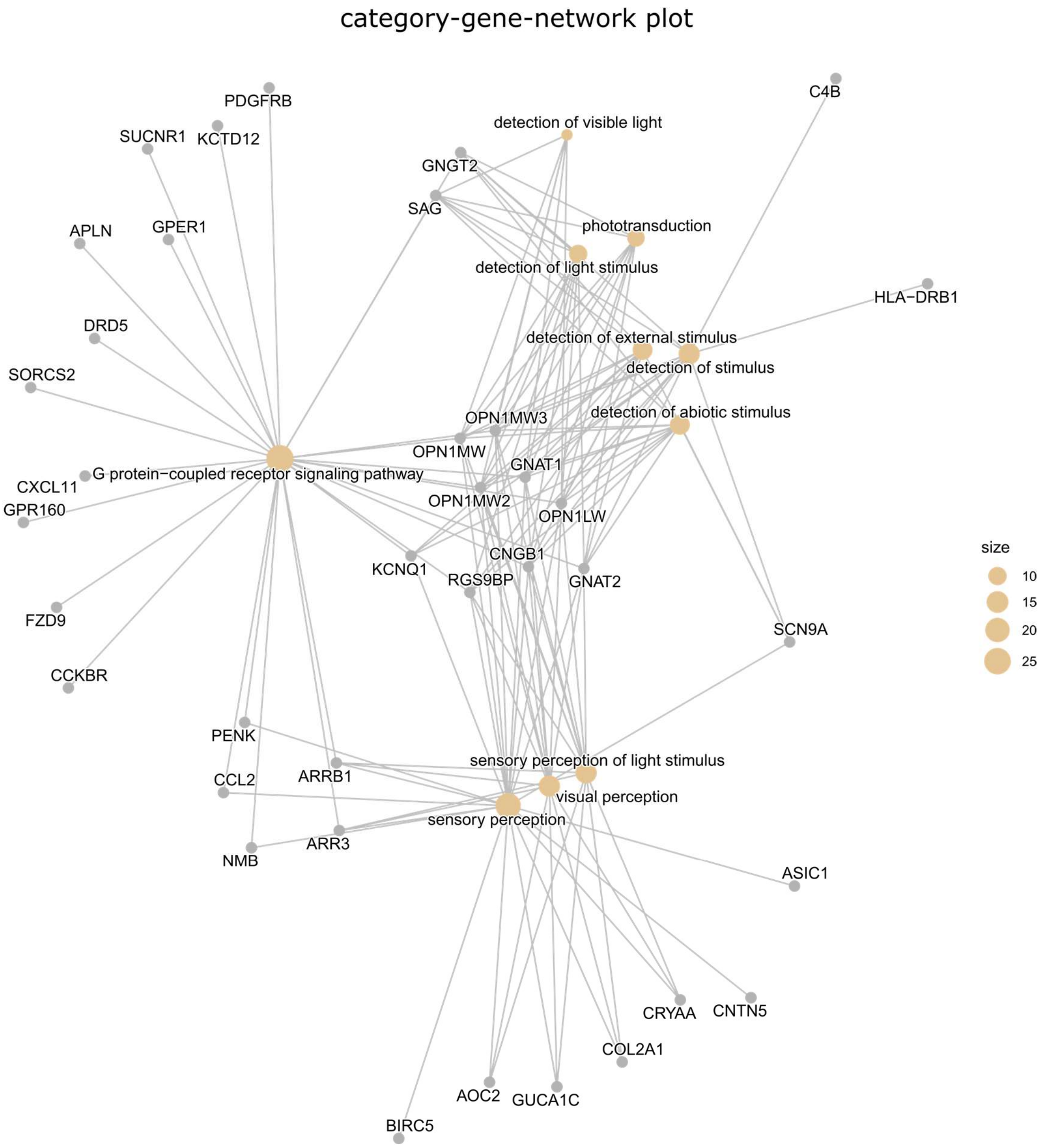

Figure S3: Category-gene network plot of GO enrichment analysis (Top 10 categories are shown)

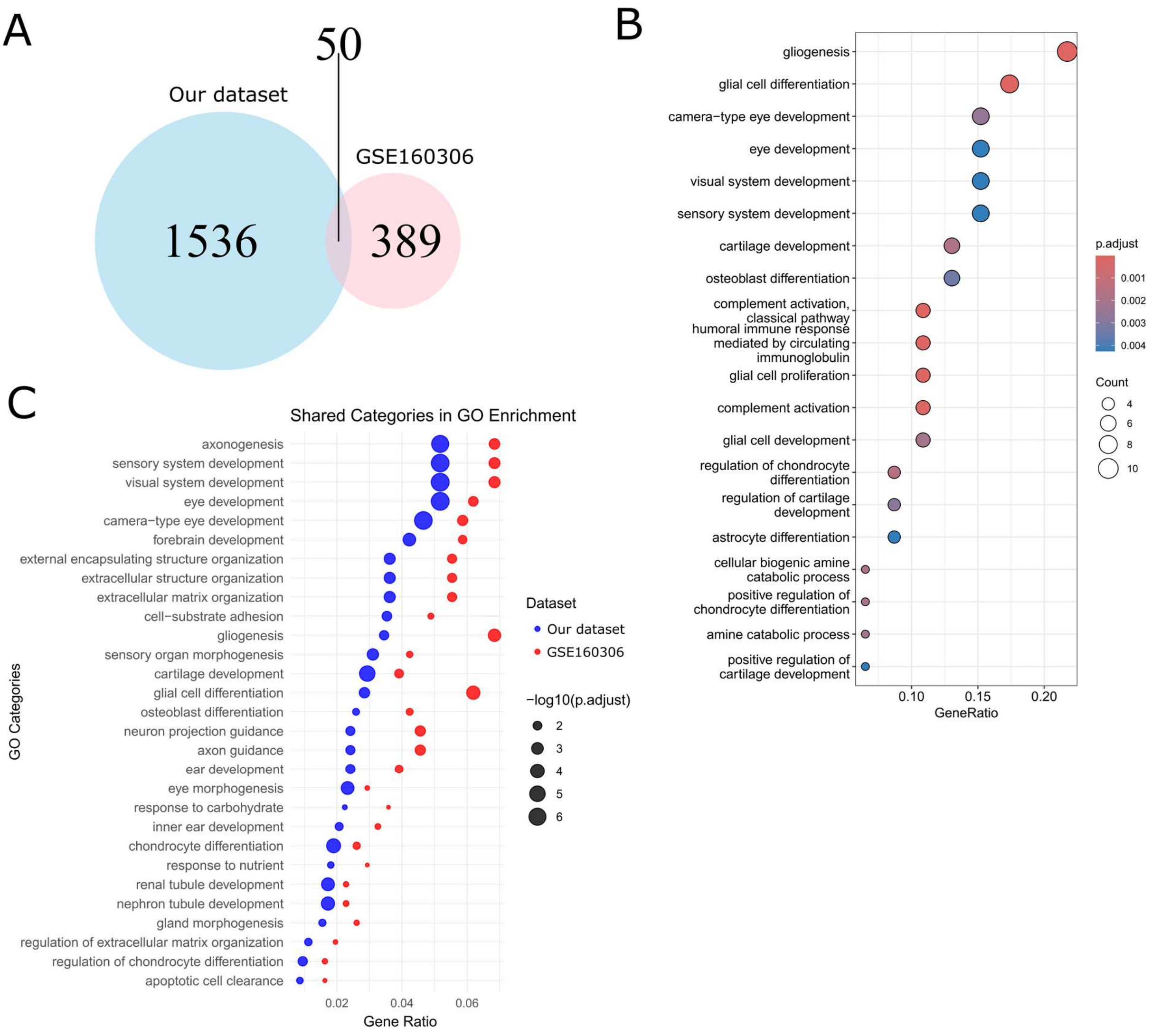

Figure S4: A) Venn diagram depicting the overlap of differentially expressed genes (p< 0.05) identified in hyperglycaemia-exposed retinal organoids (blue) and a publicly available diabetic retinopathy dataset GSE160306 (pink). The shared genes (intersection) represent common genes affected in both datasets. **B)** Dot plot showing enriched Gene Ontology (GO) terms for the shared genes. **C)** Dot plot representing enriched shared pathways in both dataset (blue dots represent our dataset, while red dots represent GSE160306).

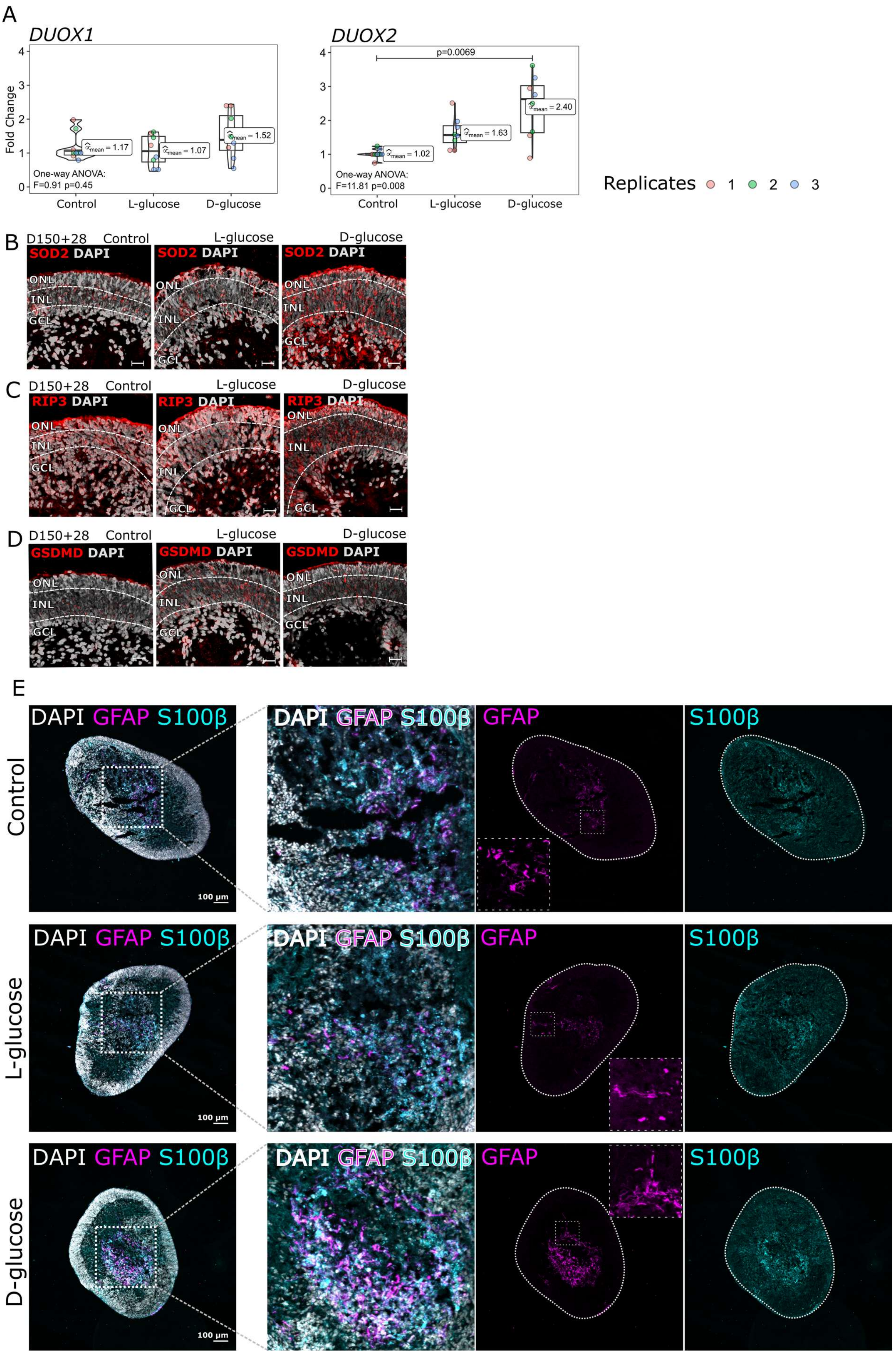
A
DUOX1
DUOX2
Fold Change
One-way ANOVA:
F=0.91 p=0.45
One-way ANOVA:
F=11.81 p=0.008
p=0.0069
Control
L-glucose
D-glucose
Replicates 1 2 3
B D150+28 Control L-glucose D-glucose
SOD2 DAPI
C D150+28 Control L-glucose D-glucose
RIP3 DAPI
D D150+28 Control L-glucose D-glucose
GSDMD DAPI
ONL
INL
GCL
E
DAPI GFAP S100β
GFAP
S100β
Control
L-glucose
D-glucose
100 µm

Figure S5: A) RT-qPCR analysis of *DUOX1* and *DUOX2* expression in late-stage (D150+28) retinal organoids treated with control, L-glucose, or D-glucose. **(B–E)** Immunofluorescence staining of retinal organoids (D150+28) for: **B)** oxidative stress marker SOD2, showing upregulation in D-glucose-treated organoids. Scale bar: 20 µm. **C)** necroptosis marker RIP3 (gene *RIPK3*), showing no difference between conditions. Scale bar: 20 µm. **D)** pyroptosis marker GSDMD, showing no difference between conditions. Scale bar: 20 µm. **E)** astrocyte markers GFAP and S100β, showing upregulation in D-glucose-treated organoids. Higher-magnification images highlight the pronounced extension of GFAP-positive filamentous processes. Scale bar: 100 µm.
