# Supplementary material for: Unveiling the cellular and molecular mechanisms of diabetic retinopathy with human retinal organoids
Source: Cell Death Dis. 2025 Dec 19;16(1):892. doi: 10.1038/s41419-025-08244-1 (PMC12717054; doi:10.1038/s41419-025-08244-1)
Supplement: Supplementary file 2 — Legends for Supplementary Tables [file 41419_2025_8244_MOESM2_ESM.docx]

**Supplementary table – legends**

**Table S1:** List of differentially expressed genes (p < 0.05, log2FoldChange >= 1) between Control group and D-glucose group.

**Table S2:** List of overlapping differentially expressed genes (p < 0.05) between our dataset and GSE160306.

**Table S3:** List of qPCR primers used in this study.

**Table S4:** List of antibodies used in this study.

**Table S5:** Detailed results of one-way ANOVA and post-hoc tests (Tukey’s or Games–Howell, as appropriate) for all quantitative comparisons presented in the main and supplementary figures.
